# Unsatisfactory Laws

**Published:** 1890-02

**Authors:** 


### Editorial.

### UNSATISFACTORY LAWS.

In an editorial in the September number of this journal stress was laid on the necessity of more efficient laws in the various States for the protection of reputable dentistry. In order to add emphasis to our remarks, the ruling in a recent case in New Hampshire was cited. This is the sentence in which the reference occurred:

"In view of the fact that there is some doubt existing in the minds of the profession regarding the constitutionality of laws governing the practice of dentistry and of medicine, and because of the recent decision of the Supreme Court of New Hampshire, declaring unconstitutional the law requiring a license to practise in that State, it behooves the boards of the different States to support one another, and to adopt such uniform action as not to trust the contest of the constitutionality of their laws in the courts, or they may find themselves without any law, which would be a serious retrogression."

A correspondent takes exception to this unadorned statement, on the ground that it is misleading, and he intimates that when writing that article we were probably not aware of all the facts in the case. As the matter is an interesting one and important to every dentist in the country, it may be well briefly to present the case.

Two men, a physician and a dentist, were indicted for practising in a certain county in New Hampshire without a license, contrary to law.

Here are the important sections of the law of that State, relating to the practice of medicine and of dentistry:

"Sect. 3. It shall not be lawful for any person who is not duly authorized to practise medicine or surgery to practise dentistry, unless such person has received a dental degree from some college, university, or medical school authorized to confer the same, or shall have obtained a license from the New Hampshire Dental Society.

"Sect. 4. Said dental society shall, at such time and in such manner as may be prescribed in its charter or by laws, elect a board of censors, consisting of three members, who shall be elected for such term as may be prescribed by the society, which board shall have authority to examine and license persons to practise dentistry. The licenses shall be recorded by the clerk of said society.

"SECT. 5. No person receiving a license as herein provided shall be authorized to practise until he shall have procured the same to be recorded by the clerk of the court in the county where he resides, if a resident of this State; if not a resident of this State, in the county where he intends to practise. Such licenses shall be recorded in a book provided for that purpose, and which shall bear the title and inscription of the medical and dental register of ——— county, and the fee for recording the same shall be fifty cents.

"SECT. 6. Each person receiving a license upon examination shall pay for the use of the society granting the same the sum of five dollars; upon diploma, one dollar.

"SECT. 7. If any person shall practise medicine, surgery, midwifery, or dentistry without being duly authorized as provided in this chapter, or after his license is revoked, he shall be punished by fine of not more than three hundred dollars for each offence.

"SECT. 8. The provisions of the preceding sections shall not apply to persons who have resided and practised their profession in the town or city of their present residence during all the time since January first, eighteen hundred and seventy-five, nor to physicians residing out of the State, when called into the State for consultation with duly licensed physicians, or to attend upon patients in the regular course of business."

The last clause is the one that gave rise to the trouble. The defendants demurred to the indictment, maintaining that a law was unconstitutional which required some to undergo an examination, to obtain a license, and to pay a fee, simply because they had changed their place of business within an arbitrary fixed period; whereas others of the same or less ability were exempted from these annoyances just because from the accident of circumstances, they had remained in one place from January 1, 1875, until the passage of this law.

The judges decided that the law was unconstitutional in that it unjustly discriminated against certain persons, and they therefore sustained the demurrer.

Our correspondent says that the eighth section was the only one concerned in the decision, and that as a whole their laws on the subject are "generally observed;" but the fact is, that this last clause is the most important of all, for it exempts from the sections preceding all those who have practised in one place since January 1, 1875, and as the judges, by their ruling, have virtually exempted those who do come within the ruling of this important section, we fail to see to whom the law may be applied.

In the course of the judges' opinion the following also occurs:

"The statute also discriminates against citizens of other States. It does not apply to persons residing and practising their profession in the same town or State from January 1, 1875, to January 1, 1879, whence persons who have resided and practised their profession continually since January 1, 1879, in the same town or city in another State are required upon removing to this State to procure a license to practise their profession."

By this it would seem that practitioners from other States cannot be compelled to procure a license in order to follow their profession in the State of New Hampshire, and the laws of this State, therefore, as they are at present, can be but little more effective than a dead letter.

Similar suits with various results have occurred in most of the States that have passed laws of this character, and such has always been the case after movements of this kind.

The necessity for these laws, and stringent laws, is apparent to every intelligent person.

Laws are generally made for the protection of society at large, not for the benefit of individuals; and the result usually is that though the welfare of the community may be advanced, yet certain persons suffer.

Granted, then, that laws protective of reputable dentistry and medicine are necessary, the organized bodies of these two professions should see to it that such laws are actually protective, so firmly made as not to allow of the entering-wedge of a lawyer's quibble.

